# No Association of Variants of the NPY-System With Obsessive-Compulsive Disorder in Children and Adolescents

**DOI:** 10.3389/fnmol.2019.00112

**Published:** 2019-05-03

**Authors:** Maximilian Franke, Annette Conzelmann, Edna Grünblatt, Anna M. Werling, Helen Spieles, Christoph Wewetzer, Andreas Warnke, Marcel Romanos, Susanne Walitza, Tobias J. Renner

**Affiliations:** ^1^Department of Child and Adolescent Psychiatry, Psychosomatics and Psychotherapy, University of Würzburg, Würzburg, Germany; ^2^Department of Child and Adolescent Psychiatry, Psychosomatics and Psychotherapy, University of Tübingen, Tübingen, Germany; ^3^University Hospital of Child and Adolescent Psychiatry, University of Zurich, Zurich, Switzerland; ^4^Department of Child and Adolescent Psychiatry and Psychotherapy, Kliniken der Stadt Köln, Cologne, Germany

**Keywords:** NPY, obsessive-compulsive, children, anxiety, neuropeptide

## Abstract

Obsessive-compulsive disorder (OCD) causes severe distress and is therefore counted by the World Health Organisation (WHO) as one of the 10 most impairing illnesses. There is evidence for a strong genetic underpinning especially in early onset OCD (eoOCD). Though several genes involved in neurotransmission have been reported as candidates, there is still a need to identify new pathways. In this study, we focussed on genetic variants of the Neuropeptide Y (NPY) system. NPY is one of the most abundant neuropeptides in the human brain with emerging evidence of capacity to modulate stress response, which is of high relevance in OCD. We focussed on tag-SNPs of *NPY* and its receptor gene *NPY1R* in a family-based approach. The sample comprised 86 patients (children and adolescents) with eoOCD with both their biological parents. However, this first study on genetic variants of the NPY-system could not confirm the association between the investigated SNPs and eoOCD. Based on the small sample size results have to be interpreted as preliminary and should be replicated in larger samples. However, also in an additional GWAS analysis in a large sample, we could not observe an associations between NPY and OCD. Overall, these preliminary results point to a minor role of NPY on the stress response of OCD.

## Introduction

Obsessive-compulsive disorder (OCD) has a life-time prevalence of 2%–3.3%, both in adults and children and has its peaks of onset around 12 years (early onset OCD, eoOCD) and in early adulthood (late onset). The occurring obsessions/compulsions are interfering significantly with the patients’ everyday life and cause severe distress and anxiety. Additionally, 75% of the patients have at least one comorbidity. In eoOCD these are in particular attention deficit hyperactivity disorder, major depression and anxiety disorders (Fireman et al., 2001). OCD is the fourth most psychiatric disorder and due to its frequently severe impact on affected patients’ lives, the World Health Organisation (WHO) counts OCD to the 10 most impairing illnesses (Karno et al., 1988; Weissman et al., 1994; Lopez and Murray, 1998).

Evidence for a strong genetic component in the development of OCD derives from twin and family genetic studies as well as segregation analyses. Prevalence in first-degree relatives of OCD patients is about four times increased and even about eight times higher in eoOCD patients (Pauls et al., 1995; Alsobrook et al., 1999; Hanna et al., 2005). A higher familial load of eoOCD was observed in general, suggesting a greater importance of genetic factors (Pauls et al., 1995; Nestadt et al., 2000). In the search for genetic underpinnings in OCD, mainly genes of the serotonergic system were focussed, driven by the pharmacological effectivity of selective serotonin reuptake inhibitors (SSRIs). Several studies aimed at the gene encoding the serotonin-transporter (Bengel et al., 1999). Nonetheless, study results are heterogeneous and replications and a recent meta-analysis revealed a rather low effect size for the serotonin transporter (Walitza et al., 2014). Further, evidence emerged that the glutamate system is involved in OCD and might be a potential alternative pharmacological treatment target, especially since the association with the glutamate receptor gene *SLC1A1* has been replicated in several studies (Wendland et al., 2009; Stewart et al., 2013; Grados et al., 2015). However, variance explained by the known candidates is still rather small and given the clinical complexity of OCD there is an understanding that many genes are involved in the disease’s pathogenesis.

In the search for further neuronal messengers involved, the Neuropeptide Y (NPY) is a highly interesting candidate (Tatemoto et al., 1982). It is one of the most abundant neuropeptides in the human brain and has multiple regulating effects in the nervous system. NPY, long known as a neuropeptide modulating feeding behavior and energy homeostasis (Morton and Schwartz, 2001), has been reported influencing neuronal processes relevant in psychiatric disorders. For instance, there is a rising evidence that the NPY-system including the NPY-receptors is involved in the development of alcohol and drug dependency (NPY1R), stress coping (NPY1R, NPY2R, NPY5R) and anxiolysis (NPY1R, NPY2R; Gerald et al., 1996; Movafagh et al., 2006; Hirsch and Zukowska, 2012; Pedragosa-Badia et al., 2013). Especially regulation of stress and anxiety levels are crucial elements in the development and maintenance of OCD. Patients affected by OCD suffer fear and simultaneously triggered stress when they experience the feeling “*something is not in order*.” NPY is released in many brain areas that participate in stress response. These are, for example, the adrenergic and noradrenergic (NA) neurons of the brainstem, the corticotropin-releasing factor (CRF)-neurons of the nucleus paraventricularis, the amygdala and the hypothalamus as well as an impact on the hypothalamic-pituitary-adrenal (HPA)-axis (Heilig, 2004; Alldredge, 2010). The NPY1R-receptor was shown to modulate anxious behavior and stress which can be reversed by NPY administration (Kormos and Gaszner, 2013). Interestingly, there is also an interaction of NPY and the serotonergic system (Diksic and Young, 2001; Pittenger and Bloch, 2014). SSRI administration leads to a higher NPY release in stressed depressive mice and a mediating role of NPY on SSRI effects is discussed (Caberlotto et al., 1998; Redrobe et al., 2005; Christiansen et al., 2011). This interplay of SSRI and NPY showed for depression might be of relevance in OCD as well, especially due to the high comorbidity rate of the two illnesses (Torres et al., 2016).

Therefore, in this molecular genetic study, we aimed at the NPY-system as a messenger system with potential influence on the pathogenesis of eoOCD. Due to their previously reported functions in the regulation of anxiety and stress, we focussed on *NPY* and its receptor *NPY1R* in a family-based approach. Genetic variants of *NPY* and *NPY1R* were genotyped in a German family-based sample. The present study is the first investigating genes of the NPY-system in eoOCD.

## Materials and Methods

### Subjects

All patients were recruited at the Department of Child and Adolescent Psychiatry, Psychosomatics and Psychotherapy, University Hospital Würzburg, Germany. Patients and parents were all of Caucasian descent and agreed to participate in the study. All participants and, in the case of minors, their parents, gave written informed consent. The study was approved by the Ethics Committees of the University of Würzburg.

Patients were included in the study after they had fulfilled the diagnostic criteria for eoOCD according to DSM-IV (American Psychiatric Association, 2000), which was valid at the time of recruitment, and ICD-10 (Dilling, 2015). Patients and parents were interviewed separately for childrens’ psychiatric disorders with the German semi-structured clinical “Diagnostic Interview for Psychiatric Disorders in Children and Adolescents” (DIPS; Schneider et al., 1995). Subsequently, the severity of symptoms was assessed by the Childrens’ Yale-Brown Obsessive Compulsive Scale (cY-BOCS; Goodman et al., 1989; Scahill et al., 1997). Subjects with comorbid disorders were only included when OCD was the main psychiatric diagnosis. Senior clinicians or psychologists performed all interviews and ratings.

Exclusion criteria were a lifetime history of psychotic disorders, Tourette’s syndrome, autism spectrum disorders, alcohol dependence or mental retardation (IQ ≤ 70).

The sample comprised 86 patients (children and adolescents) with eoOCD and their parents. Fifty-one children were female, 35 were male. The patients’ mean age was 10.7 years (SD = 2.8) at the onset of disease in a range from 3 to 15 years. In 32 patients onset was earlier than the age of 10 years. Eleven patients had tic-disorders as comorbidities. Further comorbidities as depression, ADHD or anorexia nervosa existed frequently in the patients’ medical histories but were not clinically relevant at the time of study inclusion. The sample was part of previous genetic analyses and described further in previous publications (e.g., Walitza et al., 2008).

Based on a power analysis with alpha 0.05 and beta 0.80 (according to Neumann et al., 2014) a sample size of at least *N* = 105 would have been required to unravel significant effects. Therefore, we additionally analyzed associations of NPY and OCD with the recent GWAS by Arnold et al. (2018).

To enlarge the sample size of OCD patients we used the data of the most recent GWAS meta-analysis on OCD including 2,688 individuals affected by OCD and 7,037 controls by the Psychiatric Genomics Consortium, and searched the results for each of the SNPs investigated in our study. The GWAS by the Psychiatric Genomics Consortium comprised 2,688 individuals affected by OCD and 7,037 controls. The meta-analysis comprised children as well as adults (Arnold et al., 2018).

### Gene Loci

**Figure 1:**
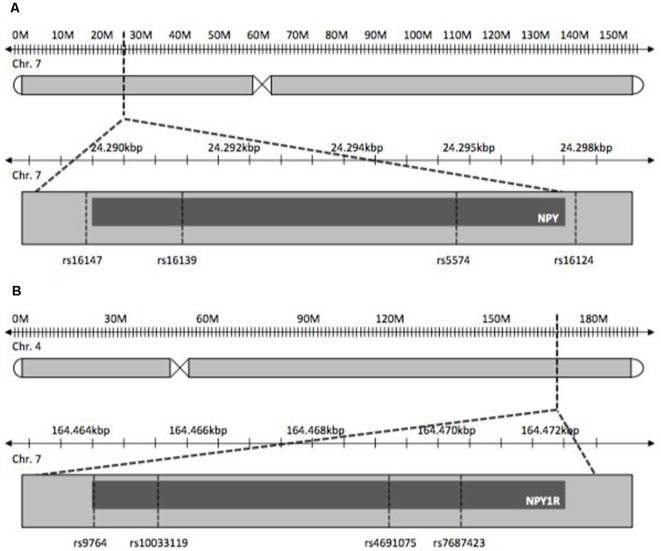
Gene loci of Neuropetide Y (NPY, **A**) and NPY-receptor (NPY1R, **B**).

*NPY* and *NPY1R* SNPs chosen for this study were tag-SNPs and previously published SNPs with reported influence on psychiatric disorders. The latter were studied with regard to stress-related diseases, as OCD is postulated to be, like depression, ADHD and obesity (Peterson et al., 2001; Tiwari et al., 2013). Tag-SNPs were determined using HaploView^®^ to cover both genes completely on the basis of SNP data provided by the International HapMap-Project (International HapMap Consortium, 2003; Barrett et al., 2005; Figure 1). Tagger settings included a minor allele frequency >0.1 and *r*^2^ = 0.9. Additionally, SNPs known from previously published studies pertaining to other disorders were included in the tagging process. For *NPY*, SNPs rs5574, rs16124, rs16139, rs16147 were determined, and SNPs rs9764, rs4691075, rs7687423 and rs10033119 were found for *NPY1R*.

### Genotyping

Genomic DNA was extracted from whole blood following standard protocols. DNA was amplified by standard PCR using specific reverse- and forward-primers for each of the eight SNPs. After amplification genotypes were determined by enzymatic digestion and gel-electrophoresis. Further detailed information on primers and procedures is available upon request.

### Statistics

Association between the included markers of *NPY* and *NPY1R* and eoOCD was tested by the Transmission Disequilibrium Test (TDT; Spielman et al., 1993). TDT was performed for all eight SNPs using the software FamHAP^®^ (Herold and Becker, 2008). A *p*-value < 0.05 was defined as the significance level. Each of the trios was tested for the hypothesis that their tested gene variants of either: (1) *NPY* or (2) *NPY1R* are associated with eoOCD. The examination pattern used for this analysis was a genotype-wise model considering every heterozygous parental genotype separately and corresponds to a test of the global null-hypothesis of transmission equilibrium of both alleles in every parental genotype. All eight SNPs were checked for Mendelian Errors which were exclusion criteria. Moreover, all parental genotype distributions were tested for Hardy-Weinberg equilibrium (HWI).

## Results

In one of the *NPY1R*-SNPs, rs4691074, occurred a deviation from the HWI (*p* = 0.04). The remaining seven SNPs were unremarkable (*p* > 0.05). No transmission disequilibrium was observed for the *NPY*-SNPs. The *p*-values exceeded the defined significance level α (Table 1). For rs5574, 34 heterogeneous parental couples could be examined ($\chi_{\left(1\right)}^{2}$ = 1.058; *p* = 0.303), for rs16124, 85 ($\chi_{\left(1\right)}^{2}$ = 0.576; *p* = 0.448), for rs16139, 12 ($\chi_{\left(1\right)}^{2}$ = 0.333; *p* = 0.564) and for rs16147, 76 ($\chi_{\left(1\right)}^{2}$ = 0.842; *p* = 0.359). Moreover, no transmission disequilibrium could be assessed for the *NPY1R*-SNPs. For rs9764, 72 heterogeneous parental couples could be analyzed ($\chi_{\left(1\right)}^{2}$ = 0.000; *p* = 1.000), for rs4691075, 33 ($\chi_{\left(1\right)}^{2}$ = 1.485; *p* = 0.223), for rs7687423, 89 ($\chi_{\left(1\right)}^{2}$ = 0.101; *p* = 0.75) and for rs10033119, 14 ($\chi_{\left(1\right)}^{2}$ = 0.000; *p* = 1.000).

**Table 1:** Transmission Disequilibrium Test (TDT) results.

SNP	Gene	Allele	T	NT	$\chi_{\left(1\right)}^{2}$	*p*TDT
rs5574	NPY	2	20	14	1.059	0.303
		4	14	20		
rs16124	NPY	3	39	46	0.576	0.448
		4	46	39		
rs16139	NPY	2	7	5	0.333	0.564
		4	5	7		
rs16147	NPY	2	34	42	0.842	0.359
		4	42	34		
rs9764	NPY1R	1	36	36	0.000	1.000
		3	36	36		
rs4691075	NPY1R	1	20	13	1.485	0.223
		3	13	20		
rs7687423	NPY1R	2	46	43	0.101	0.750
		4	43	46		
rs10033119	NPY1R	2	7	7	0.000	1.000
		4	7	7		

*Note: Column 1 lists the SNP names, column 2 the correspondent gene, column 3 shows the present allele: 1 equal to adenine, 2 equal to cytosine, 3 equal to guanine, 4 equal to thymine. Column 4 shows the number of transmissions (T) for each gene, column 5 shows the number of non-transmitted (NT) alleles. Column 6 represents the chi square value, column 7 the p-value*.

The GWAS meta-analysis also showed no association of any *NPY*- nor *NPY1R*-SNPs with regard to the development of OCD (Table 2).

**Table 2:** Meta-analysis results.

Chr.	SNP	bp	A1	A2	Info	OR	SE	*p*
7	rs5574	24329133	T	C	0.9750	0.967152	0.0949	0.7248
7	rs16124	24331799	T	G	0.9735	1.00944	0.0335	0.7802
7	rs16139	24324879	T	C	0.9737	0.983144	0.0335	0.6112
7	rs16147	24323410	T	C	0.9720	1.01572	0.0335	0.6421
4	rs9764	164245405	T	C	1.0030	1.03345	0.0382	0.3881
4	rs4691075	164249485	T	C	0.9948	0.937255	0.0504	0.1991
4	rs7687423	164250797	A	G	0.9800	1.001	0.0347	0.9766
4	rs10033119	164245854	A	G	0.8709	0.962809	0.0851	0.6562

*Note: Column 1 lists the respective chromosomes (hg 19), column 2 the correspondent marker name, column 3 shows the base pair location (hg 19), column 4 shows the reference allele for OR (may or may not be minor allele), column 5 the alternative allele. Column 6 represents the imputation information score, column 7 the Odds ratio for the effect of the A1 allele, column 8 the standard error of the log(OR) and column 9 the p-value for the association test in the meta-analysis*.

## Discussion

This is the first molecular genetic study on potential functional variants of the candidate gene *NPY* and its receptor *NPY1R* in eoOCD. In this study, association was not detected. Thus, we could not confirm a major role of the NPY system in OCD with childhood onset.

As our number of trios was smaller than postulated by the power-analysis, the negative outcome might foremost be due to the sample size. That is why we used the data of the most recent and aforementioned GWAS meta-analysis on OCD by the Psychiatric Genomics Consortium, and searched the results for the investigated SNPs. Though our sample was part of this meta-analysis, a potential effect over a large sample including different ages of onset might have been observable. However, also this meta-analysis showed no association of any *NPY*- nor *NPY1R*-SNPs (Arnold et al., 2018). Our study had been designed as a family-based study to avoid stratification effects, nonetheless, a case-control study, which had not been performed so far with regard to the studied gene loci, would be highly interesting.

However, the NPY system, which is involved in stress response, might still be of relevance in OCD subgroups, although undetected due to the study design. The selected SNPs were either already known from earlier studies to other stress-related psychiatric disorders or tag-SNPs that were selected out of the HapMap data with the help of the programme Haploview^®^ (Barrett et al., 2005) in order to cover *NPY* and *NPY1R*. Nonetheless, the selected SNPs are infrequent in the population, thus statistically evaluable transmissions in the sample were rather small.

Reflecting the NPY-system, other effectors like the Y_2_-receptor and the Y_5_-receptor might also be valuable targets in OCD. The Y_1_-receptor was the first choice for this study due to its various anxiolytic and stress-reducing effects and its wide spread dissemination in stress regulating brain regions. Anxious behavior and stress can be reversed with NPY administration (Kormos and Gaszner, 2013). Nevertheless, the influence of the remaining receptors should not be excluded* a priori*. Especially the Y_5_-receptor shares a similar effect spectrum with the tested Y_1_ (Kormos and Gaszner, 2013). Even though its role is not fully understood yet, an effect on the fear and stress system seems to be obvious and further research worthwhile. The Y_2_-receptor, however, could indirectly influence the delicate equilibrium of neurotransmission with its impact on the release of other neurotransmitters (Upadhya et al., 2009; Kormos and Gaszner, 2013). A reduced inhibition of glutamate release, for example, could be a correlate for the increased thalamic and striatal glutamate activity in untreated OCD-children and could explain the upregulation of Y_2_-receptors in a state of anxiety in the mouse model (Leckman et al., 1997; Upadhya et al., 2009).

Our sample consists of children and adolescents only, resembling an OCD subgroup with early onset and a significant number of children with an onset before the age of 10 years. A stronger impact of genetic factors is reported for eoOCD and our sample might differ from adult samples regarding genetic underpinnings and impact of life-events (Nestadt et al., 2000; Walitza et al., 2008). The responsivity of the NPY system after stress exposure during the development has been shown in animal models and could be of higher relevance in failed coping and development of OCD in adults (Serova et al., 2017; Yam et al., 2017).

Moreover, comorbidities as e.g., ADHD or depression were accepted in this study. Nonetheless, comorbidities in OCD might indicate distinct neurobiological OCD subgroups with divergent etiologies (Taurines et al., 2010). An analysis reflecting the comorbidities was not applicable due to the sample size. Especially an analysis regarding comorbidity with depression would be of interest (Caberlotto et al., 1998; Redrobe et al., 2005; Christiansen et al., 2011).

Due to the sample size, we were not able to examine an influence of gender, which was reported for other candidate genes in previous studies. In the meta-analyses of the glutamate transporter gene *SLC1A1*, the SNP rs12682807 was found associated only in male probands (Thiele et al., 1998; Arnold et al., 2006; Wendland et al., 2009). Other genetic publications also stated gender differences (Thiele et al., 1998; Dickel et al., 2006; Canals et al., 2012). Gender differences are also found in the clinical perspective with the prevalence of subclinical OCD-symptoms twice as high in boys than in girls (Canals et al., 2012). Furthermore, the two sexes differ in the clinical manifestation which comprises an earlier age of onset as well as a higher prevalence of symptoms belonging to the entity of symmetry and ordering among males and increased symptoms of cleanliness and washing among females (Bogetto et al., 1999; Stewart et al., 2007).

Though the sample size was rather small for a genetic study, regarding eoOCD it has a considerable size. Since the sample was collected in one facility, it provides a clear stringency regarding a precisely defined phenotype and restrictive exclusion of severe comorbid disorders to assure the predominance of definite OCD in contrast to obsessive-compulsive symptoms in other psychiatric disorders.

In conclusion, our family-based study on genetic variants of NPY and NPY1R could not confirm association with OCD with childhood onset correlating with the outcomes for adult NPY and OCD by Altemus et al. (1999). Unfortunately, there is overall a paucity of studies on NPY and OCD and we can only add another hint for non-existing significant effects. We only know so far that NPY is involved in stress and other anxiety disorders. Therefore, we hope that we could deliver with our study a contribution to the question about an association of NPY with OCD. The major impact of our study is that we could show in our sample—and comprehending the forementioned GWAS-data—that NPY effects on OCD should be very small or not present. The very impact of our study is a sample consisting of early onset patients and therefore of fundamental impact on the database in this specific subgroup. Moreover, the outcome has a meaning for the understanding of the stress response in OCD in general which might be different from other anxiety disorders with an association to NPY. Indeed, in DSM V OCD is no longer listed under anxiety disorders. If our null finding is replicated in larger studies, we might conclude that NPY has no effect on the stress response in OCD and probably might also be a less valuable target for pharmacological research in OCD.

## Author Contributions

All authors certify that they have participated sufficiently in the work to take public responsibility for the content. TR was responsible for conception and design of the study. TR and MF carried out data acquisition and analysis. TR, MF and AC were mainly involved in drafting the article. TR, MF, AC, EG, AMW, SW, CW, MR, AW and HS contributed to data interpretation and manuscript draft and revision.

## Conflict of Interest Statement

The authors declare that the research was conducted in the absence of any commercial or financial relationships that could be construed as a potential conflict of interest.
